# Supplementary material for: Cockayne Syndrome Patient iPSC-Derived Brain Organoids and Neurospheres Show Early Transcriptional Dysregulation of Biological Processes Associated with Brain Development and Metabolism
Source: Cells. 2024 Mar 28;13(7):591. doi: 10.3390/cells13070591 (PMC11011893; doi:10.3390/cells13070591)
Supplement: Supplementary file 1 [file cells-13-00591-s001.zip › organoids-2917974-supplementary - sub/Supplementary Figures S1-S12 with Legends.pdf]

Cockayne syndrome patient iPSC-derived brain organoids and neurospheres show early transcriptional dysregulation of biological processes associated with brain development and metabolism.

Organoids

Leon-Phillip Szepanowski[1], Wasco Wruck[1], Julia Kapr, Andrea Rossi, Ellen Fritsche, Jean Krutmann and James Adjaye[1,*]

[1]Institute for Stem Cell Research and Regenerative Medicine, University Hospital Düsseldorf, Moorenstrasse 5, D-40225 Duesseldorf, Germany

*Correspondence: James.Adjaye@med.uni-duesseldorf.de

Leon-Phillip.Szepanowski@IUF-Duesseldorf.de

Supplementary

A

	B4	CS789	IUFi001
**Cell Type**	Fetal Foreskin Fibroblast	Dermal Fibroblast	Dermal Fibroblast
**Provided By**	ISRM Düsseldorf	IUF - Leibniz Research Institute for Environmental Medicine	
**Donor Age**	Neonatal	10 months	3 years
**Sex**	♂	♂	♀
**Reprogramming**	Viral	Episomal	Episomal
***ERCC6* Mutation**	None	Exon 10/R683X	Exon 5/K377X; Exon15/R857X
**CS Severity**	None	COFS	CS Type I

B

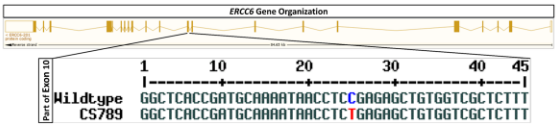

C

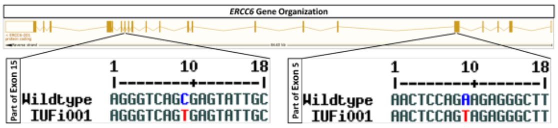

D

	Cerebrooculofacioskeletal (COFS) syndrome	Cockayne Syndrome Type II	Cockayne Syndrome Type I	Cockayne Syndrome Type III	UV-sensitive Syndrome
**Onset**	Fetal	At birth	First 2 years	3-4 years	Varying
**Diagnosed at**	Birth	Infancy	Childhood	Early teens	Childhood/Adulthood
**Life Expectancy**	Infancy	5-6 Years	16 years	30 years	Normal?
**Suggestive Findings**	Athrogryphosis<br>Prenatal growth failure<br>Prenatal Microcephaly<br>Congenital Cataracts **or** Congenital Microphthalmia	**Major Criteria:**<br>Postnatal growth failure; Progressive Microcephaly; Neurologic Dysfunction with developmental delay; White matter dysmyelination; Cerebellar Atrophy; Intracranial calcifications<br>**Minor Criteria:**<br>Cutaneous Photosensitivity; Demyelinating peripheral neuropathy; Pigmentary Retinopathy; Cataracts; Sensorineural Hearing loss; Enamel Hypoplasia; Tooth Anomalies; Cachectic Dwarfism			
**Diagnosis**	Multigene panel **or** Comprehensive genomic testing				

Supplementary Figure S1. Used cell lines and clinical information about Cockayne Syndrome (A) General information about the cell lines used in this work. (B) Schematic depiction of the ERCC6 mutation found in the CS789 iPSC line. (C) Schematic depiction of the ERCC6 mutation found in the IUFi001 iPSC line. (D) Table showing clinical features and diagnostic criteria of different types of Cockayne Syndrome.

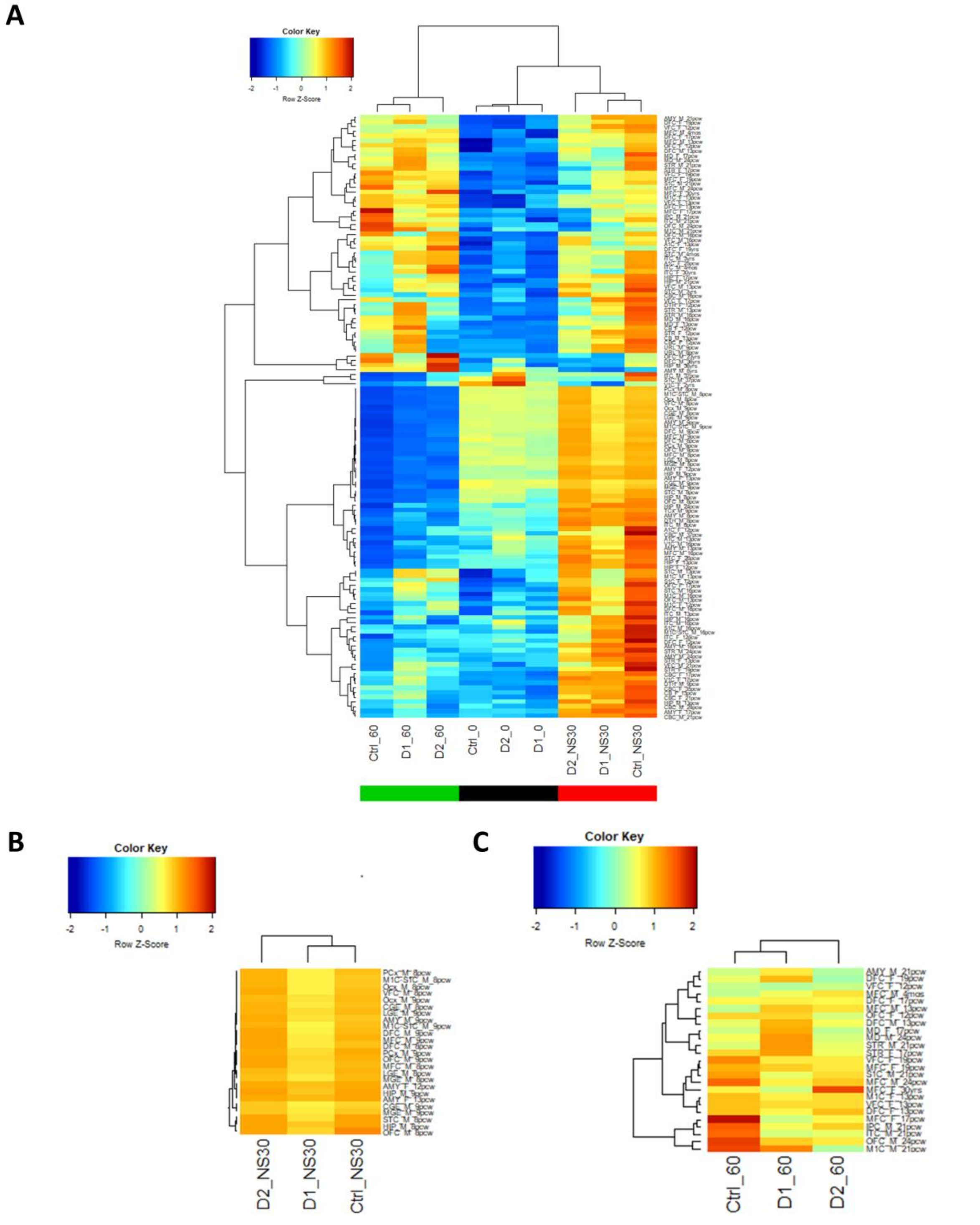

Supplementary Figure S2. Determination of developmental age of day 30 neurospheres and day 60 cortical organoids (A) Heat map of enrichment scores of transcriptomic data from control and CS iPSCs, neurospheres and organoids in comparison with the Allen Brain Atlas (ABA) human developmental Brain NGS data (https://www.brainspan.org/). (B) Region of highest consensus for the day 30 neurospheres extracted from A. (C) Region of highest consensus for the day 60 organoids extracted from A.

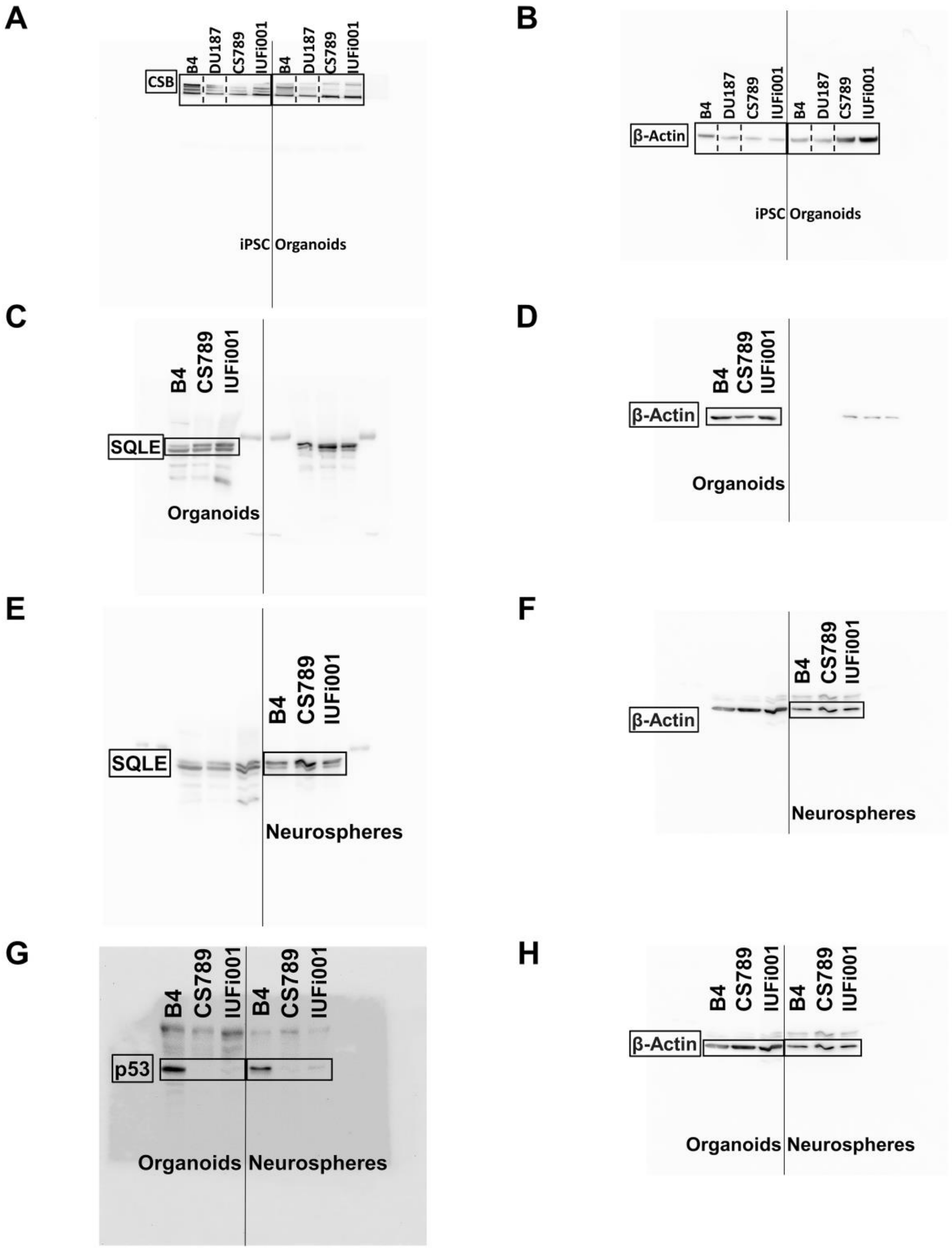

Supplementary Figure S3. Full Western Blot for CSB, SQLE, p53 and Beta-Actin protein in Day 0 iPSCs, Day 30 NS and Day 60 COs (A) Western Blot analysis for full-length CSB protein in B4, DU187, CS789 and IUFi001 iPSCs and COs. The cell line DU187 was not included in this article. (B) Western Blot for beta-Actin protein in B4, DU187, CS789 and IUFi001 iPSCs and COs. The cell line DU187 was not included in this article. (C) Western Blot analysis for SQLE protein in B4, CS789 and IUFi001 COs. (D) Western Blot analysis for beta-Actin protein in B4, CS789 and IUFi001 COs. (E) Western Blot analysis for SQLE protein in B4, CS789 and IUFi001 NS. (F) Western Blot analysis for beta-Actin protein in B4, CS789 and IUFi001 NS. (G) Western Blot analysis for p53 protein in B4, CS789 and IUFi001 NS and COs. (H) Western Blot analysis for beta-Actin protein in B4, CS789 and IUFi001 NS and COs. (B, D, F, H) Blot of the housekeeping gene used to quantify the respective blot in A, C, E and G on the left side of the figure. (Black outlines indicate parts of the image utilized to prepare the main figures. Dotted lines in A and B indicate where the DU187 line was excised from the image.)

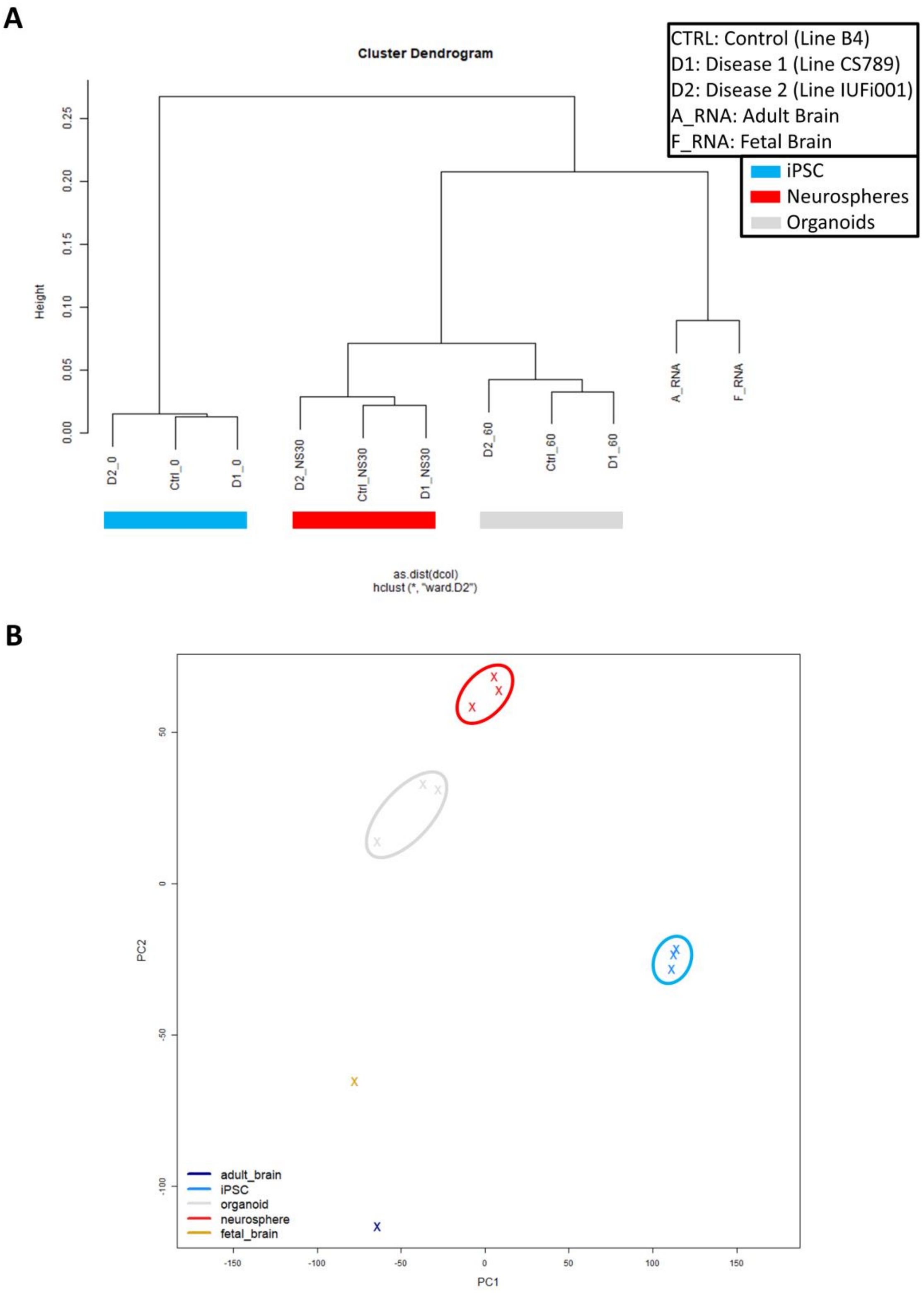

Supplementary Figure S4. Quality control of next generation sequencing data (A) Dendrogram obtained by hierarchical cluster analysis of NGS gene expression data for B4, CS789 and IUFi001 iPSCs, neurospheres and cerebral organoids. (B) Principal component analysis of NGS gene expression data for B4, CS789 and IUFi001 iPSCs, neurospheres and cerebral organoids.

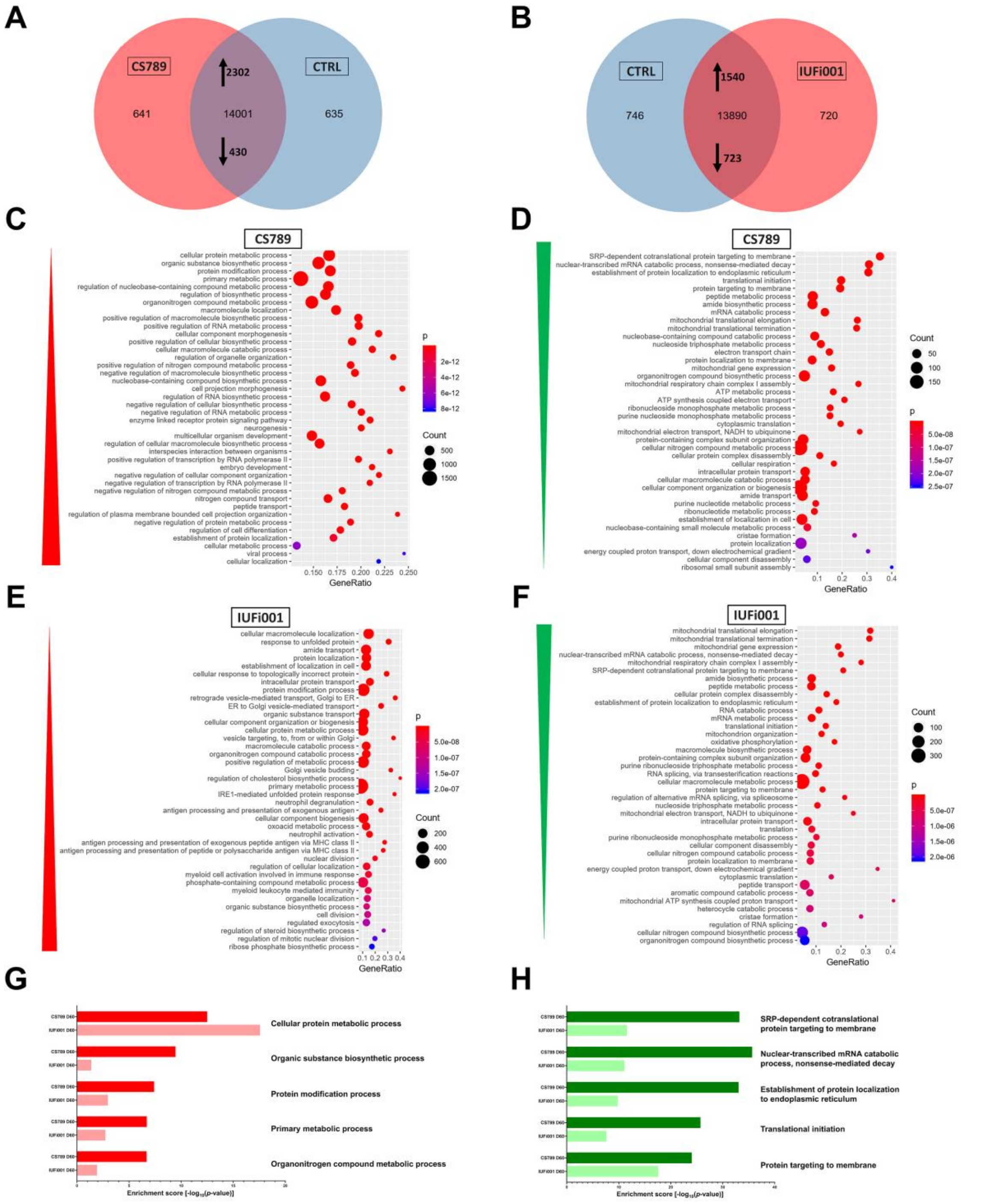

Supplementary Figure S5. Global transcriptome and associated Gene Ontology analysis of control and CS neurospheres at day 30 (A) Venn diagram showing genes expressed only in CS789 neurospheres (641), in CTRL (B4) neurospheres (635) and common to both (14001) (detection p value < 0.05). (B) Venn diagram showing genes expressed only in IUFi001 neurospheres (720), in CTRL (B4) neurospheres (746) and common to both (13890) (detection p value < 0.05). (C,D) Dot plots showing the Top 30 differentially regulated Gene Ontologies (c) in the 2302 significantly upregulated DEGs in day 30 CS789 neurospheres in comparison to CTRL (B4) (D) and in the 430 significantly downregulated DEGs in day 30 CS789 neurospheres in comparison to CTRL (B4). (E,F) Dot plots showing the Top 30 differentially regulated KEGG pathways (E) in the 1540 significantly upregulated DEGs in day 30 IUFi001 neurospheres in comparison to CTRL (B4) (F) and in the 723 significantly downregulated DEGs in day 30 IUFi001 neurospheres in comparison to CTRL (B4). (G) Bar chart of the differentially upregulated Gene Ontologies (Top 5 ranked) common between day 30 CS789 and IUFi001 neurospheres in comparison to CTRL (B4) neurospheres. (H) Bar chart of the differentially downregulated Gene Ontologies (Top 5 ranked) common between day 30 CS789 and IUFi001 neurospheres in comparison to CTRL (B4) neurospheres.

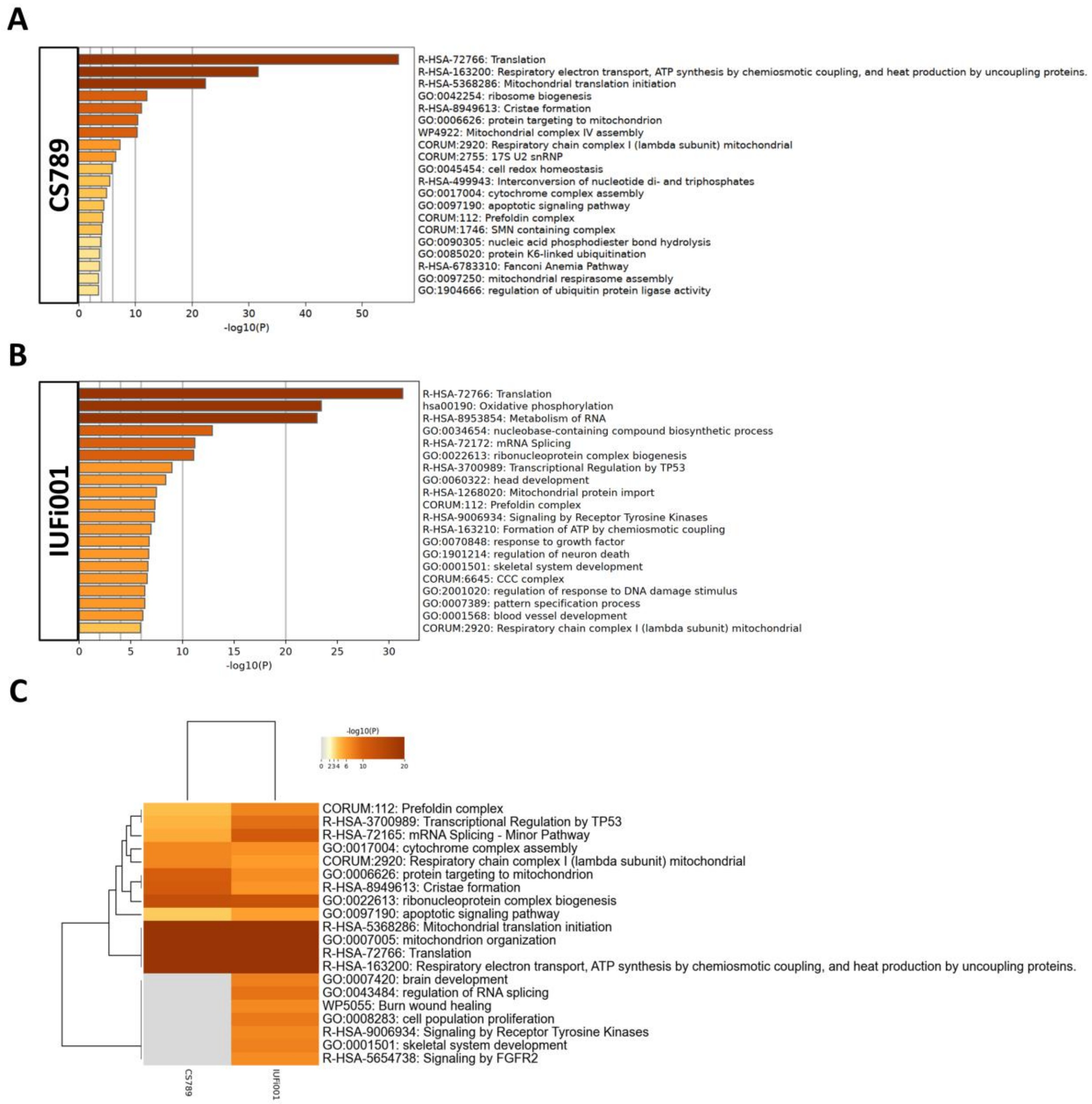

Supplementary Figure S6. Comparative transcriptome and Gene Ontology analysis of downregulated DEGs in day 30 neurospheres (A) Bar graph of the Top 20 non-redundant enrichment clusters attributable to the 430 DEGs upregulated in day 30 CS789 neurospheres in comparison to CTRL (B4). (B) Bar graph of the Top 20 non-redundant enrichment clusters attributable to the 723 DEGs downregulated in day 30 IUFi001 neurospheres. (C) Metascape-generated heatmap comparing downregulated gene-sets employed in A and B revealed i.a. GOs involved in mitochondrial translation initiation, mitochondrion organization and translation.

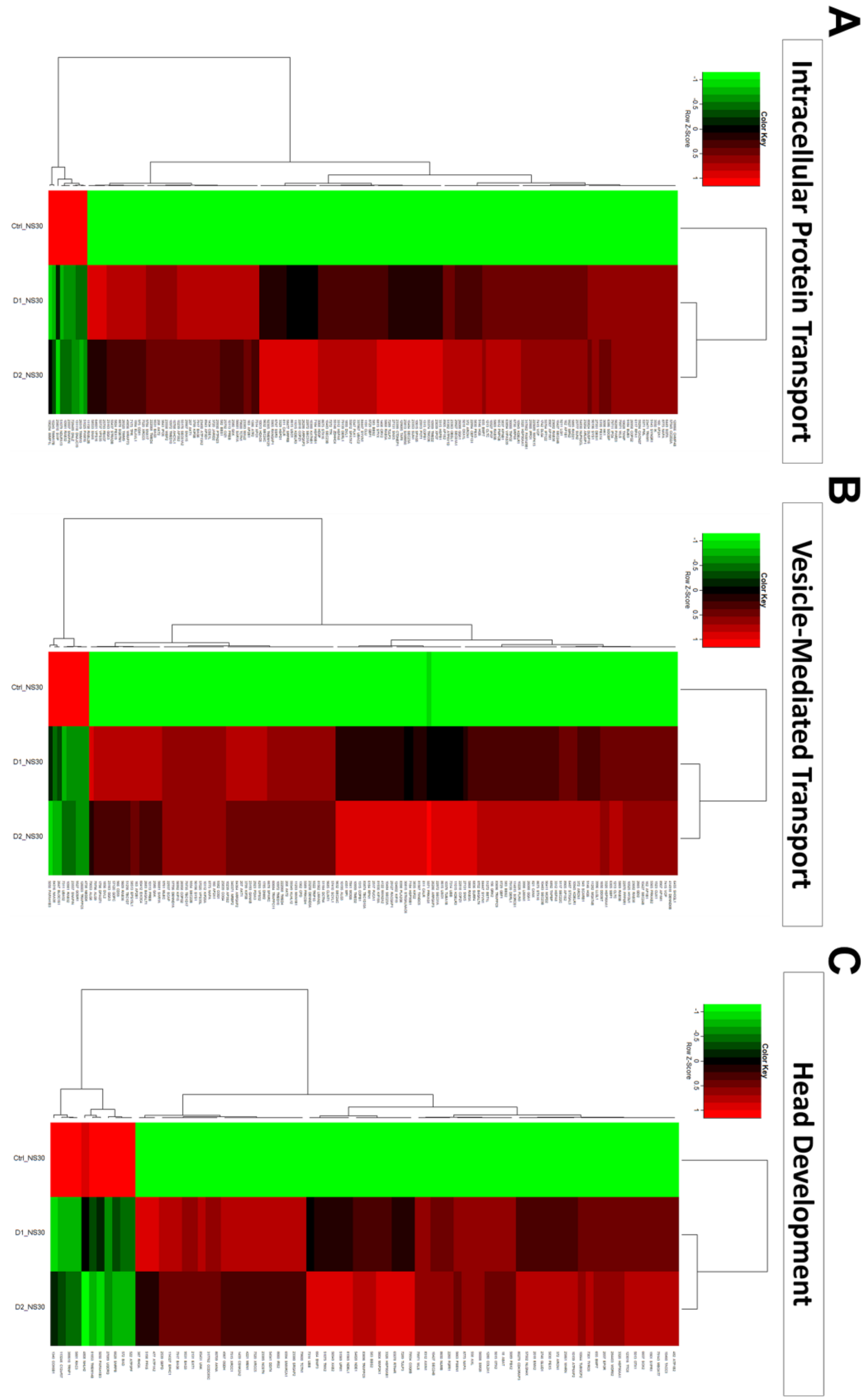

Supplementary Figure S7. Analysis of select Gene Ontologies differentially regulated in day 30 neurospheres (A) Pearson´s heatmap depicting all identified differentially regulated genes involved in intracellular protein transport common between day 30 CS789 (D1) and IUFi001 (D2) neurospheres in comparison to B4 (CTRL). (B) Pearson´s heatmap depicting all identified differentially regulated genes involved in vesicle-mediated transport common between day 30 CS789 (D1) and IUFi001 (D2) neurospheres in comparison to B4 (CTRL). (C) Pearson´s heatmap depicting all identified differentially regulated genes involved in head development common between day 30 CS789 (D1) and IUFi001 (D2) neurospheres in comparison to B4 (CTRL).

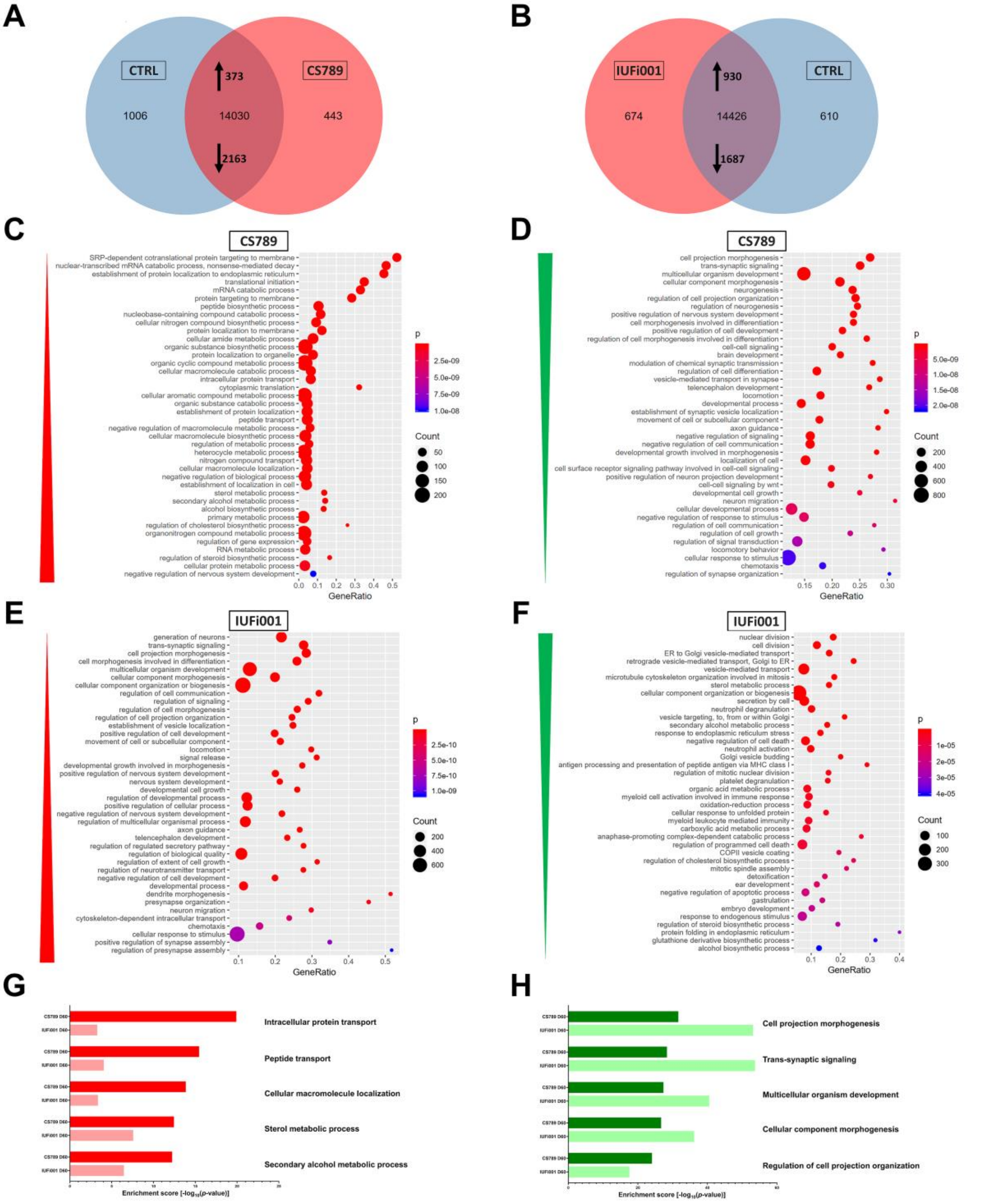

Supplementary Figure S8. Global transcriptome and associated Gene Ontology analysis of control and CS organoids at day 60 (A) Venn diagram showing genes expressed only in CS789 organoids (443), in CTRL (B4) organoids (1006) and common to both (14030) (detection p-value < 0.05). (B) Venn diagram showing genes expressed only in IUFi001 organoids (674), in CTRL (B4) organoids (610) and common to both (14426) (detection p value < 0.05). (C,D) Dot plots showing the Top 30 differentially regulated Gene Ontologies (C) in the 373 significantly upregulated DEGs in day 60 CS789 organoids in comparison to CTRL (B4) (D) and in the 2163 significantly downregulated DEGs in day 60 CS789 organoids in comparison to CTRL (B4). (E,F) Dot plots showing the Top 30 differentially regulated Gene Ontologies (E) in the 930 significantly upregulated DEGs in day 60 IUFi001 organoids in comparison to CTRL (B4) (F) and in the 1687 significantly downregulated DEGs in day 60 IUFi001 organoids in comparison to CTRL (B4). (G) Bar chart of the differentially upregulated Gene Ontologies (Top 5 ranked) common between day 60 CS789 and IUFi001 organoids in comparison to CTRL (B4) organoids. (H) Bar chart of the differentially downregulated Gene Ontologies (Top 5 ranked) common between day 60 CS789 and IUFi001 organoids in comparison to CTRL (B4) organoids.

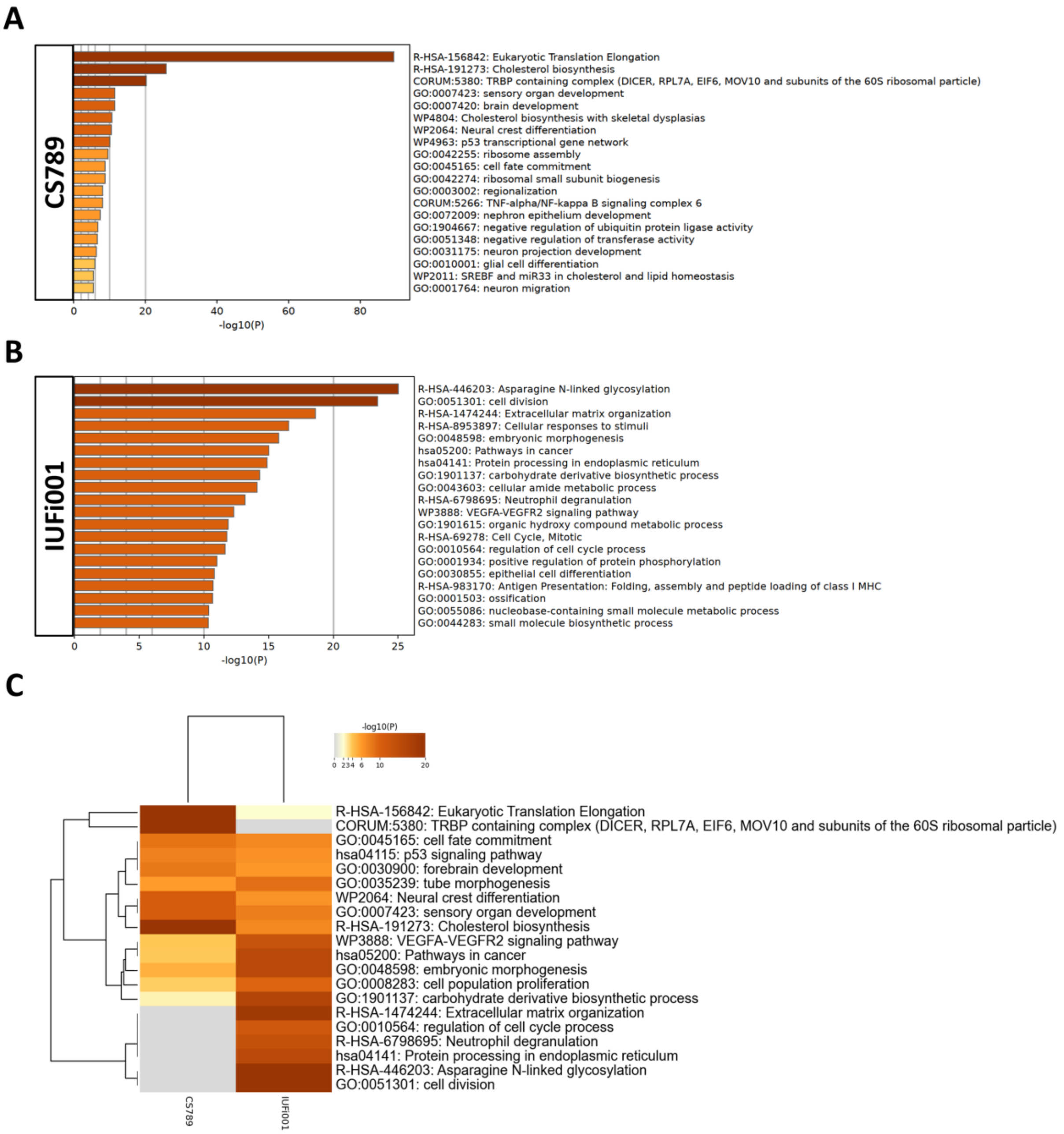

Supplementary Figure S9. Comparative transcriptome and Gene Ontology analysis of upregulated DEGs in day 60 organoids (A) Bar graph of the Top 20 non-redundant enrichment clusters attributable to the 373 DEGs upregulated in day 60 CS789 organoids in comparison to CTRL (B4). (B) Bar graph of the Top 20 non-redundant enrichment clusters attributable to the 930 DEGs upregulated in day 60 IUFi001 organoids. (C) Metascape-generated heatmap comparing upregulated gene-sets employed in A and B revealed i.a. GOs involved in forebrain development, p53-signalling pathway, cholesterol biosynthesis and VEGFA-VEGFR2 signalling pathway.

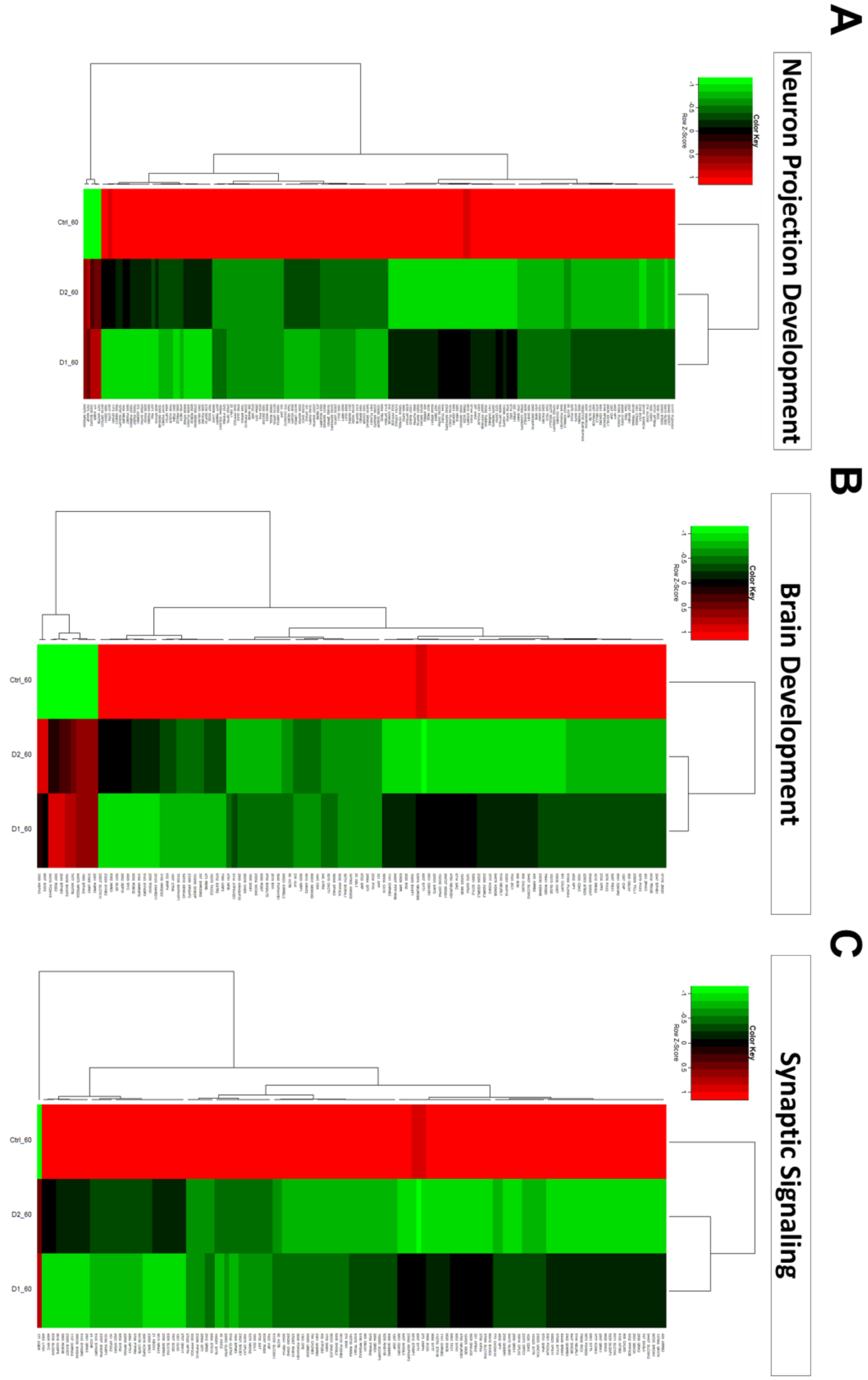

Supplementary Figure S10. Analysis of select Gene Ontologies differentially regulated in day 60 organoids (A) Pearson´s heatmap depicting all identified differentially regulated genes involved in neuron projection development common between day 60 CS789 (D1) and IUFi001 (D2) organoids in comparison to B4 (CTRL). (B) Pearson´s heatmap depicting all identified differentially regulated genes involved in brain development common between day 60 CS789 (D1) and IUFi001 (D2) organoids in comparison to B4 (CTRL). (C) Pearson´s heatmap depicting all identified differentially regulated genes involved in synaptic signalling common between day 60 CS789 (D1) and IUFi001 (D2) organoids in comparison to B4 (CTRL).

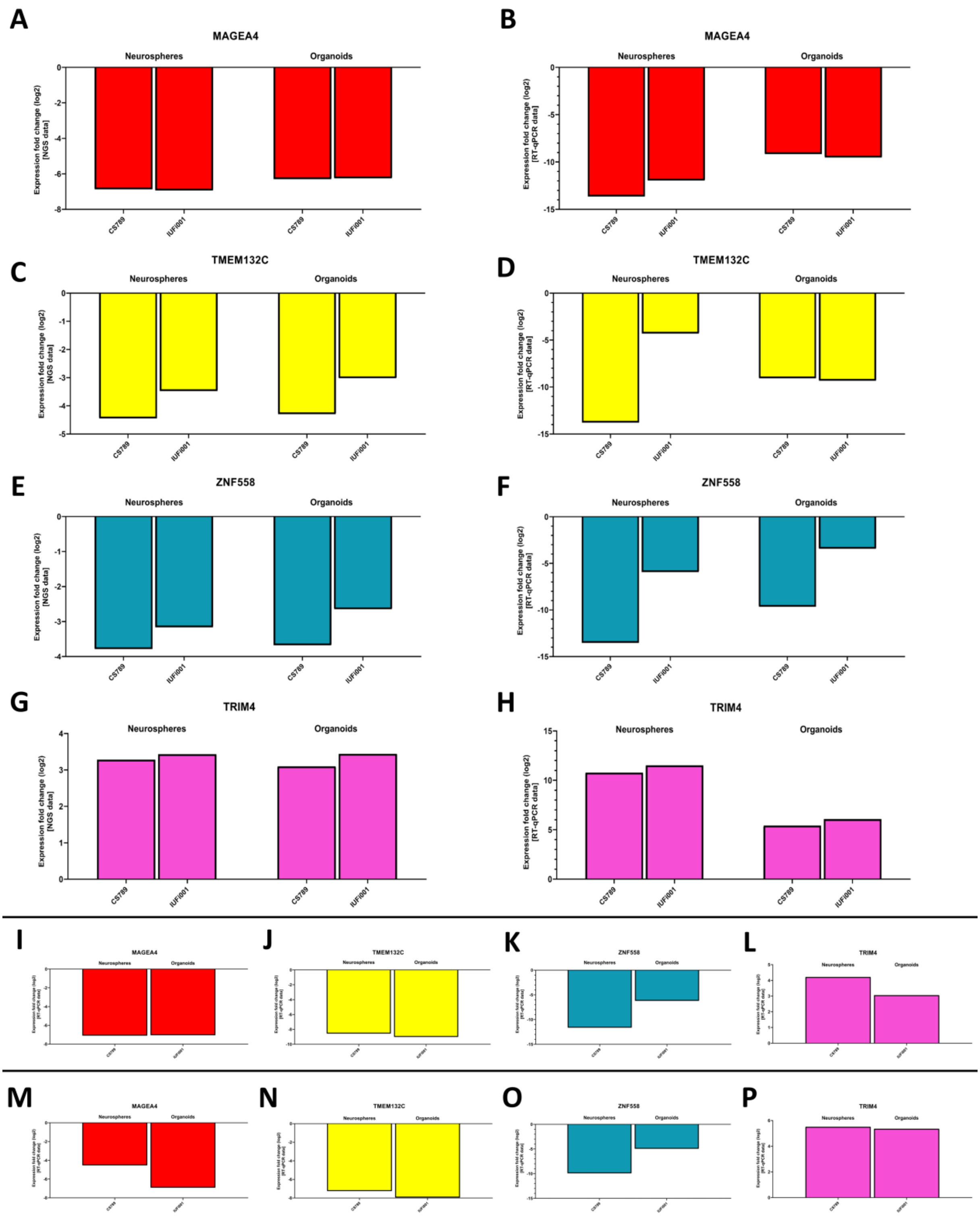

Supplementary Figure S11. RT-qPCR for MAGEA4, TMEM132C, ZNF558 and TRIM4 in Day 30 Neurospheres, two sets of Day 60 Organoids and Day 120 Organoids (A,C,E,G) Relative mRNA expression analysis of *MAGEA4* (A), *TMEM132C* (C), *ZNF558* (E), and *TRIM4* (G) in CS789 and IUFi001 day 30 neurospheres and day 60 organoids compared to CTRL (B4). (B,D,F,H) qRT-PCR analysis of *MAGEA4* (B), *TMEM132C* (D), *ZNF558* (F), and *TRIM4* (H) mRNA expression in CS789 and IUFi001 day 30 neurospheres and day 60 organoids relative to CTRL (B4). (I-L) RT-qPCR analysis of MAGEA4 (I), TMEM132C (J), ZNF558 (K) and TRIM4 (L) mRNA expression in a second set of day 60 CS789 and IUFi001 organoids relative to control organoids (B4). (M-P) RT-qPCR analysis of MAGEA4 (M), TMEM132C (N), ZNF558 (O) and TRIM4 (P) mRNA expression of day 120 CS789 and IUFi001 organoids relative to control organoids (B4).

A

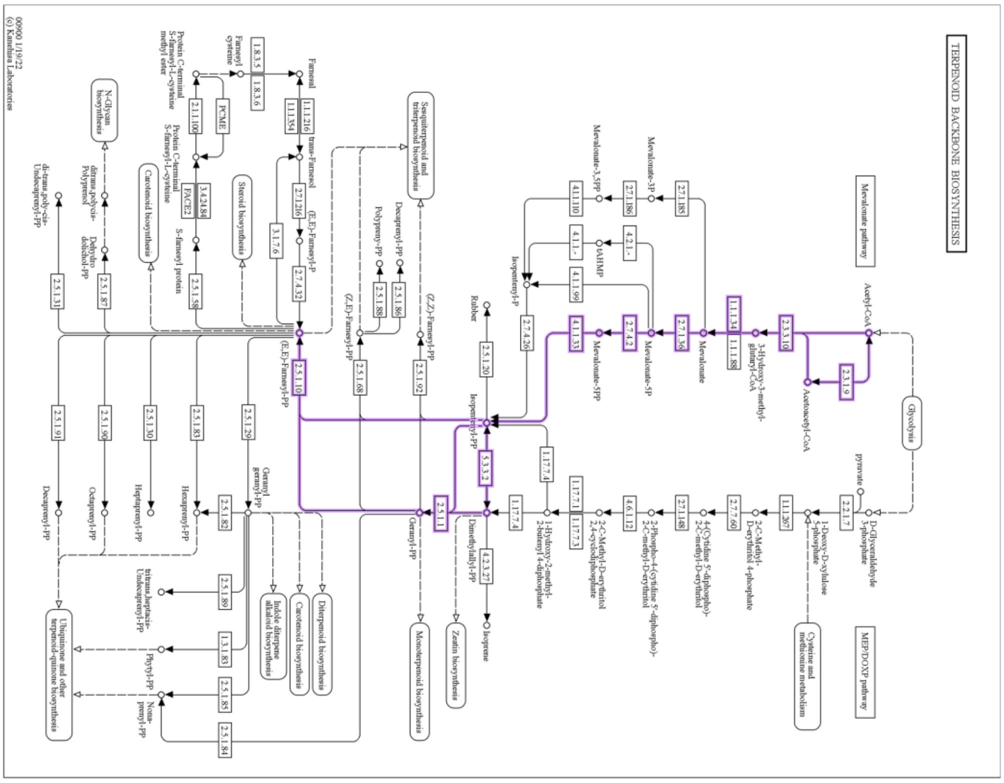

B

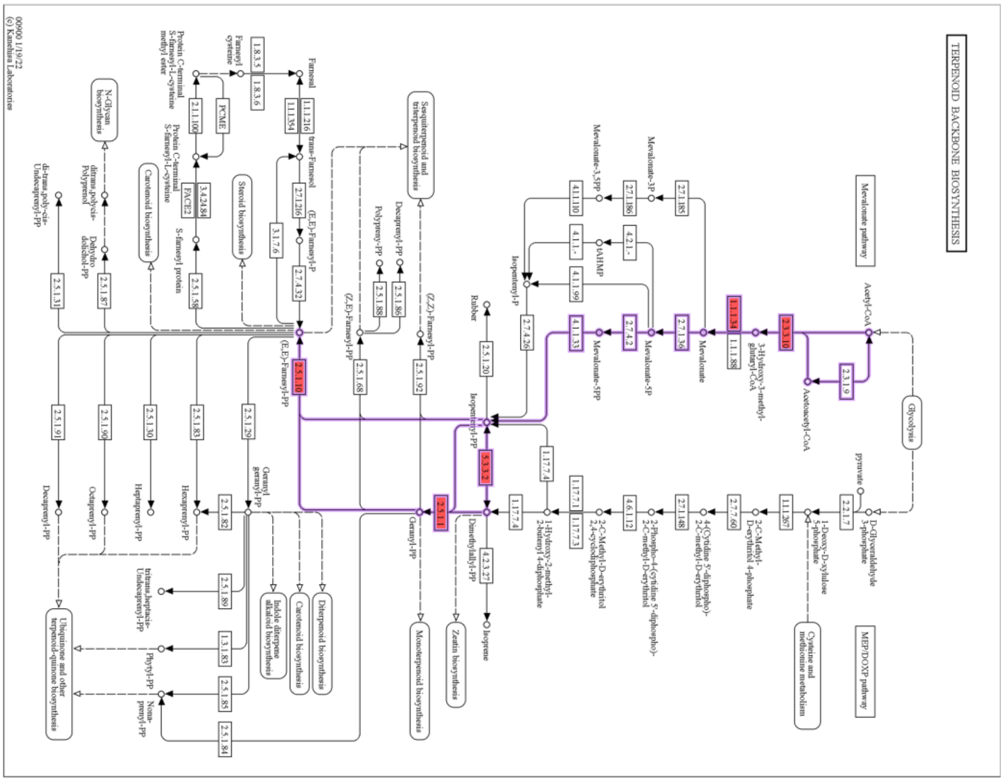

Supplementary Figure S12. Analysis of the Mevalonate pathway of the Terpenoid Backbone Biosynthesis pathway in day 30 NS and day 60 COs (A,B) Schematic of the pathway Terpenoid Back Biosynthesis with the Mevalonate pathway indicated in purple. The genes upregulated in CSB-deficient day 30 NS (A) and day 60 COs (B) are indicated in red.
