# Supplementary figures and images for: Cockayne Syndrome Patient iPSC-Derived Brain Organoids and Neurospheres Show Early Transcriptional Dysregulation of Biological Processes Associated with Brain Development and Metabolism
Source: Cells. 2024 Mar 28;13(7):591. doi: 10.3390/cells13070591 (PMC11011893; doi:10.3390/cells13070591)

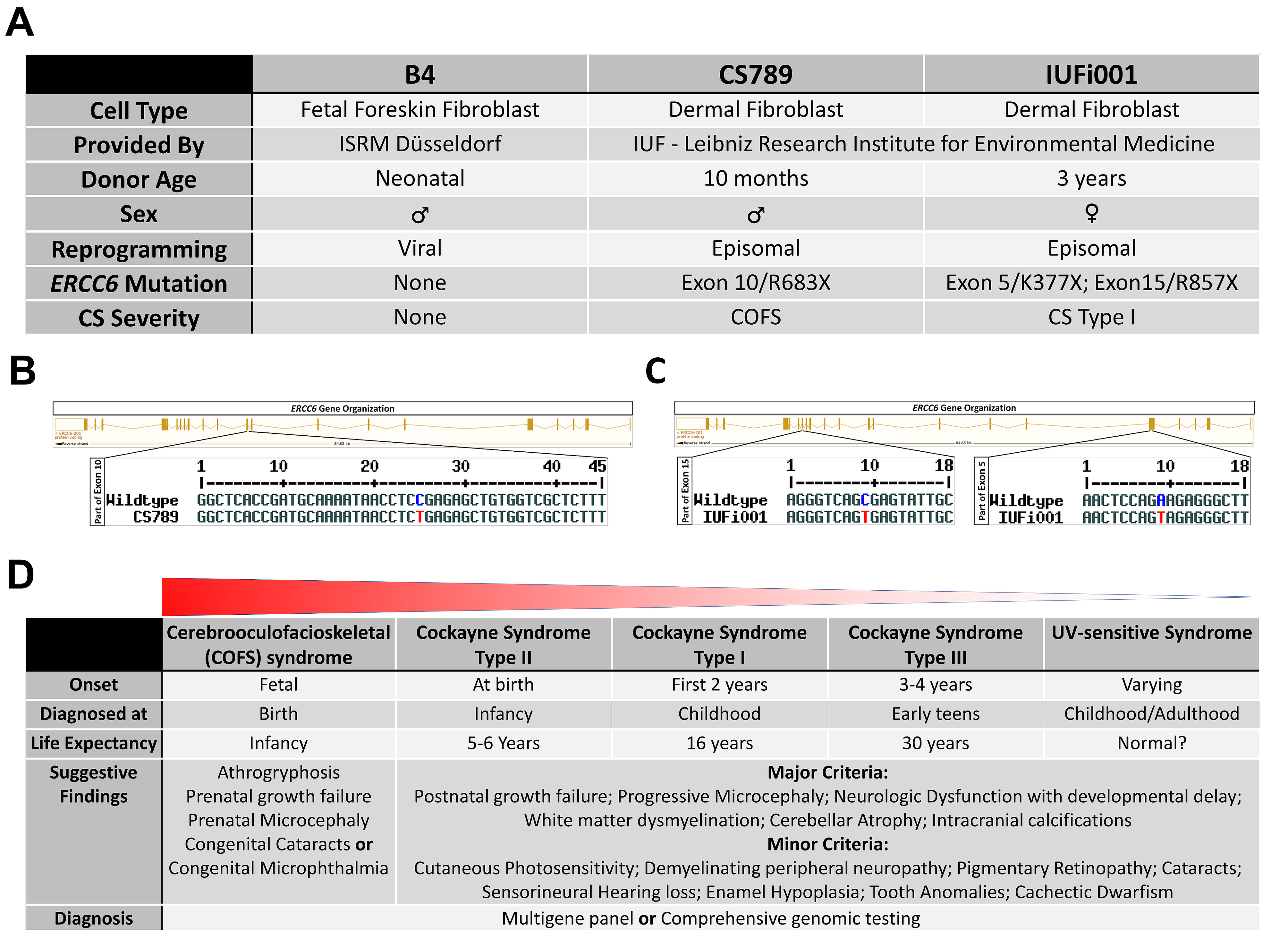

Supplement: Supplementary file 1 [file cells-13-00591-s001.zip › organoids-2917974-supplementary - sub/S1.TIF]

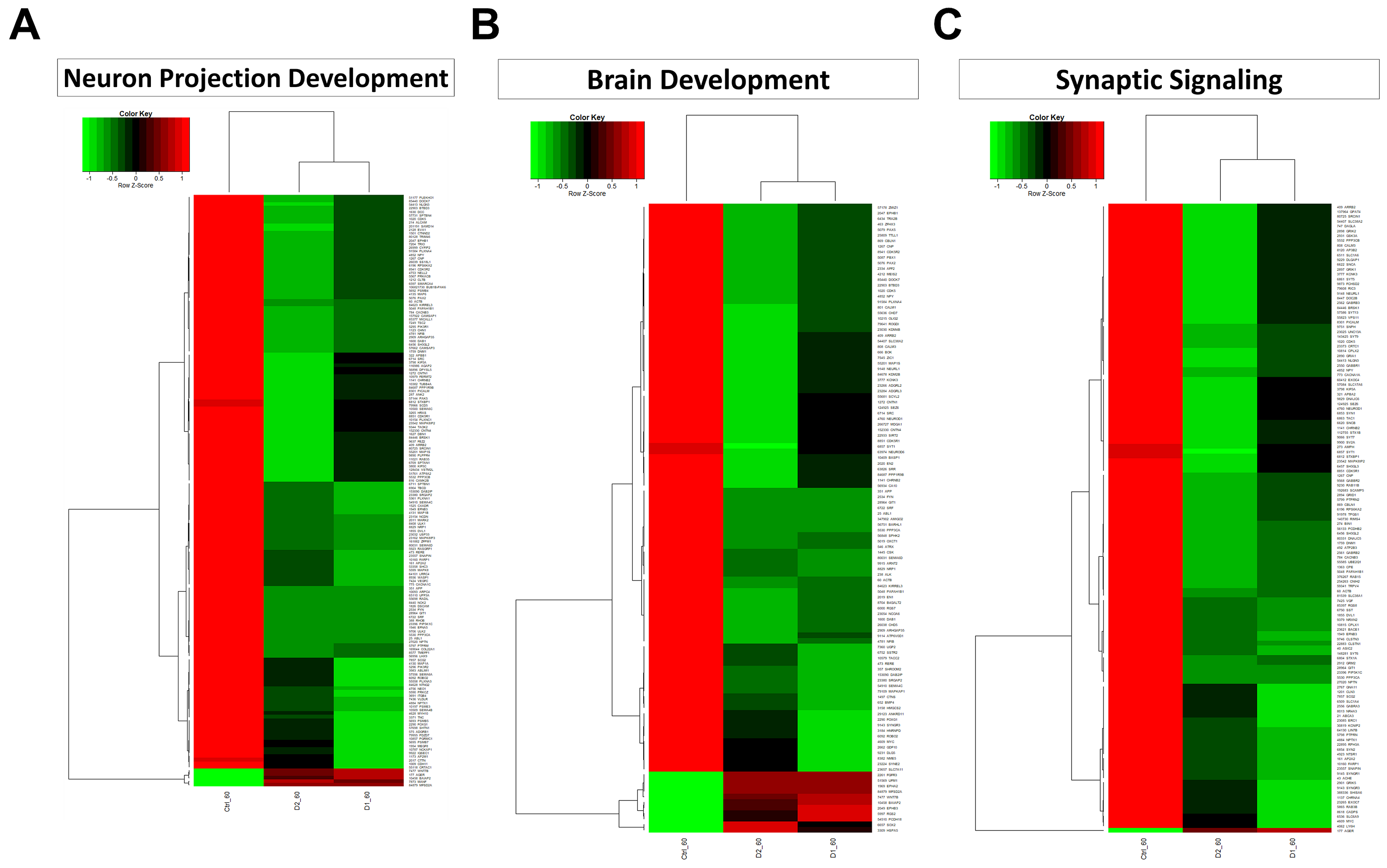

Supplement: Supplementary file 1 [file cells-13-00591-s001.zip › organoids-2917974-supplementary - sub/S10.TIF]

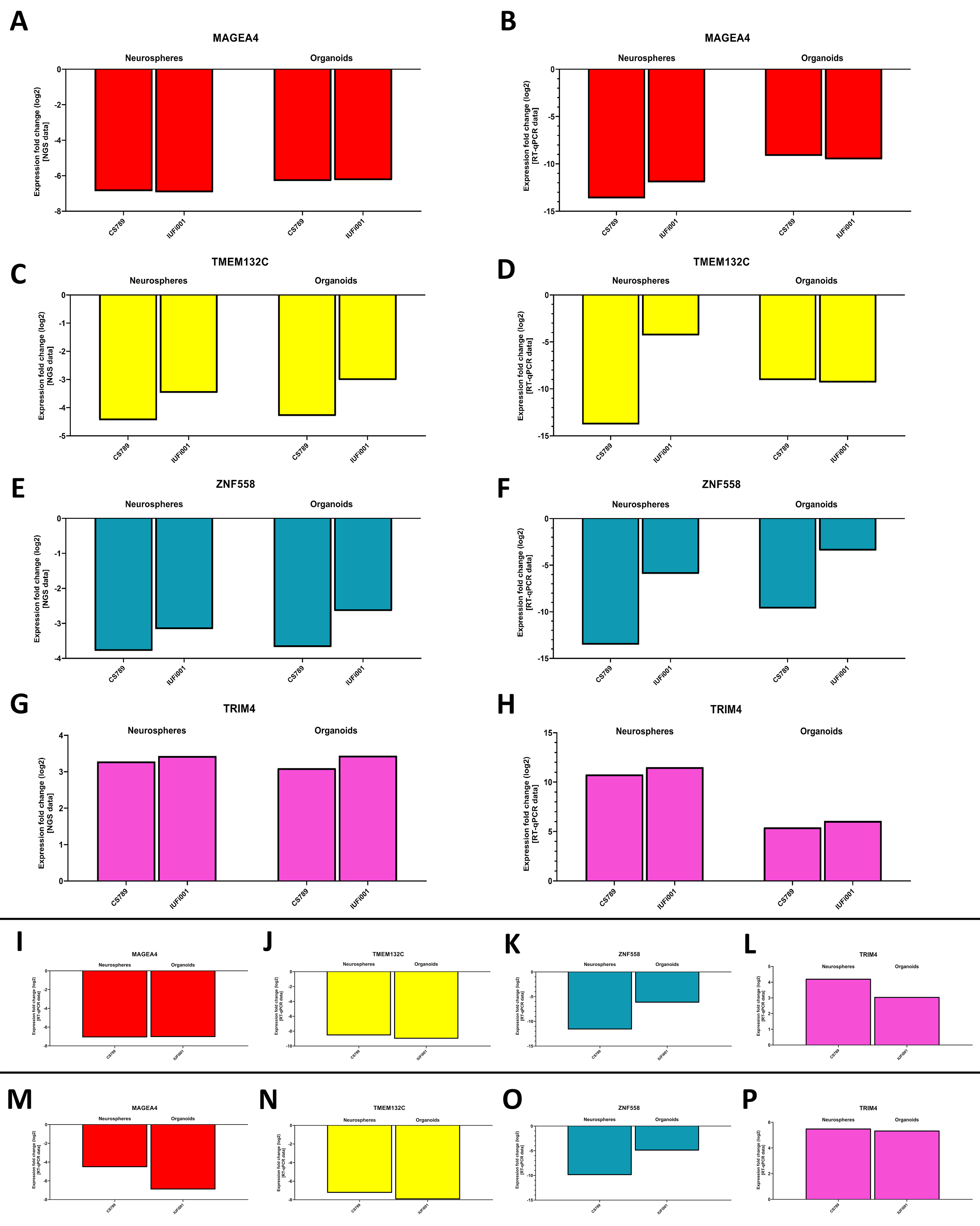

Supplement: Supplementary file 1 [file cells-13-00591-s001.zip › organoids-2917974-supplementary - sub/S11.TIF]

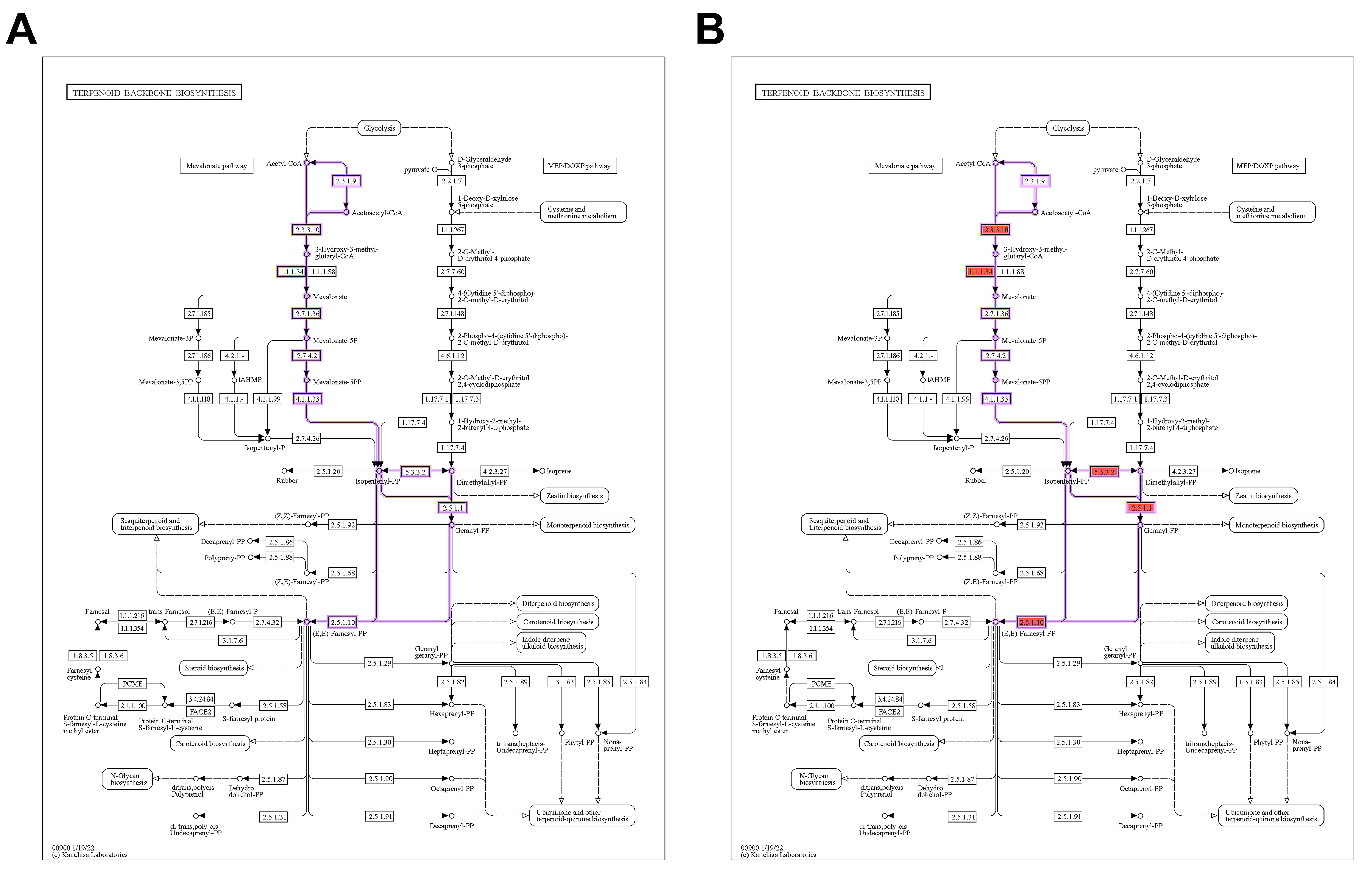

Supplement: Supplementary file 1 [file cells-13-00591-s001.zip › organoids-2917974-supplementary - sub/S12.TIF]

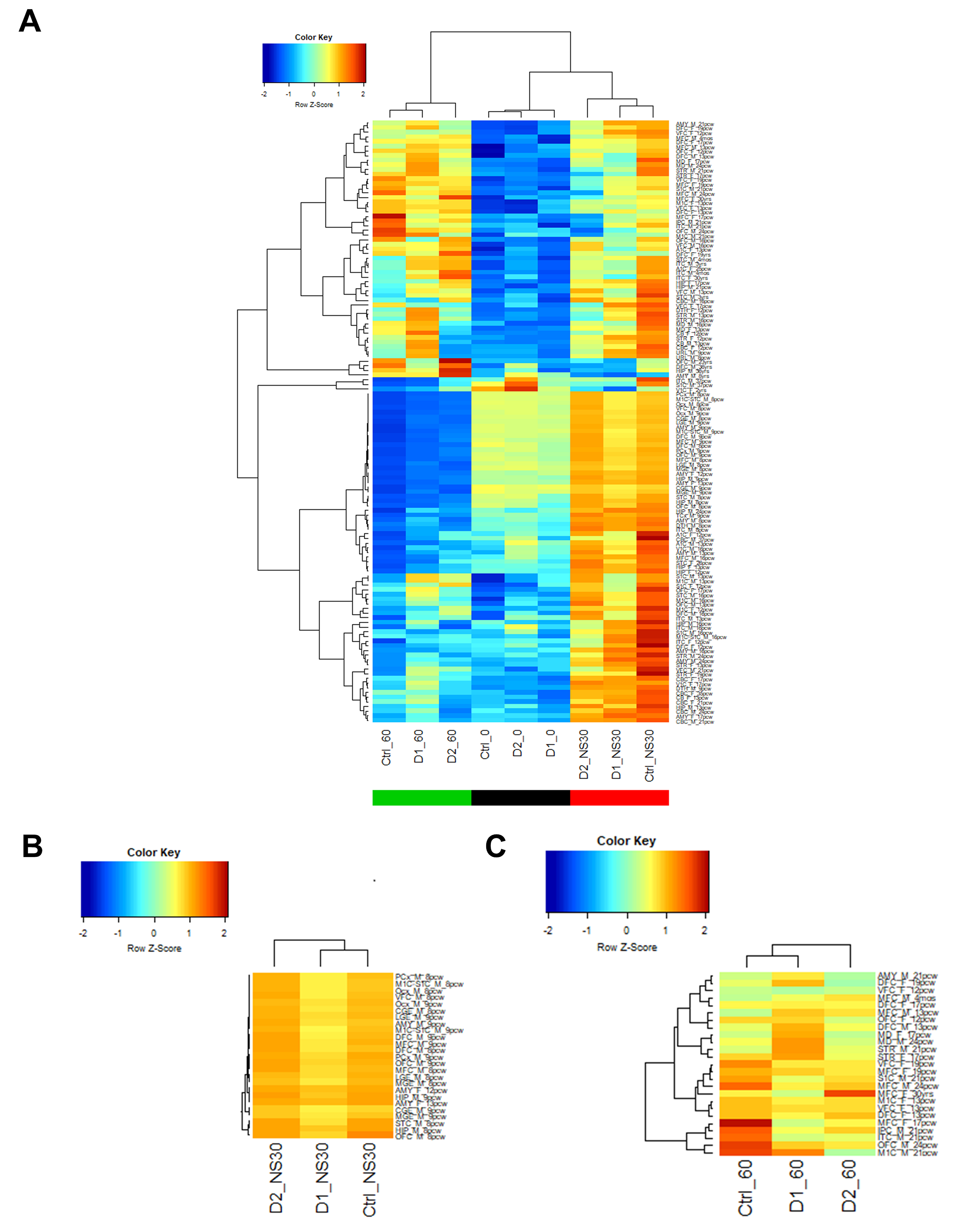

Supplement: Supplementary file 1 [file cells-13-00591-s001.zip › organoids-2917974-supplementary - sub/S2.TIF]

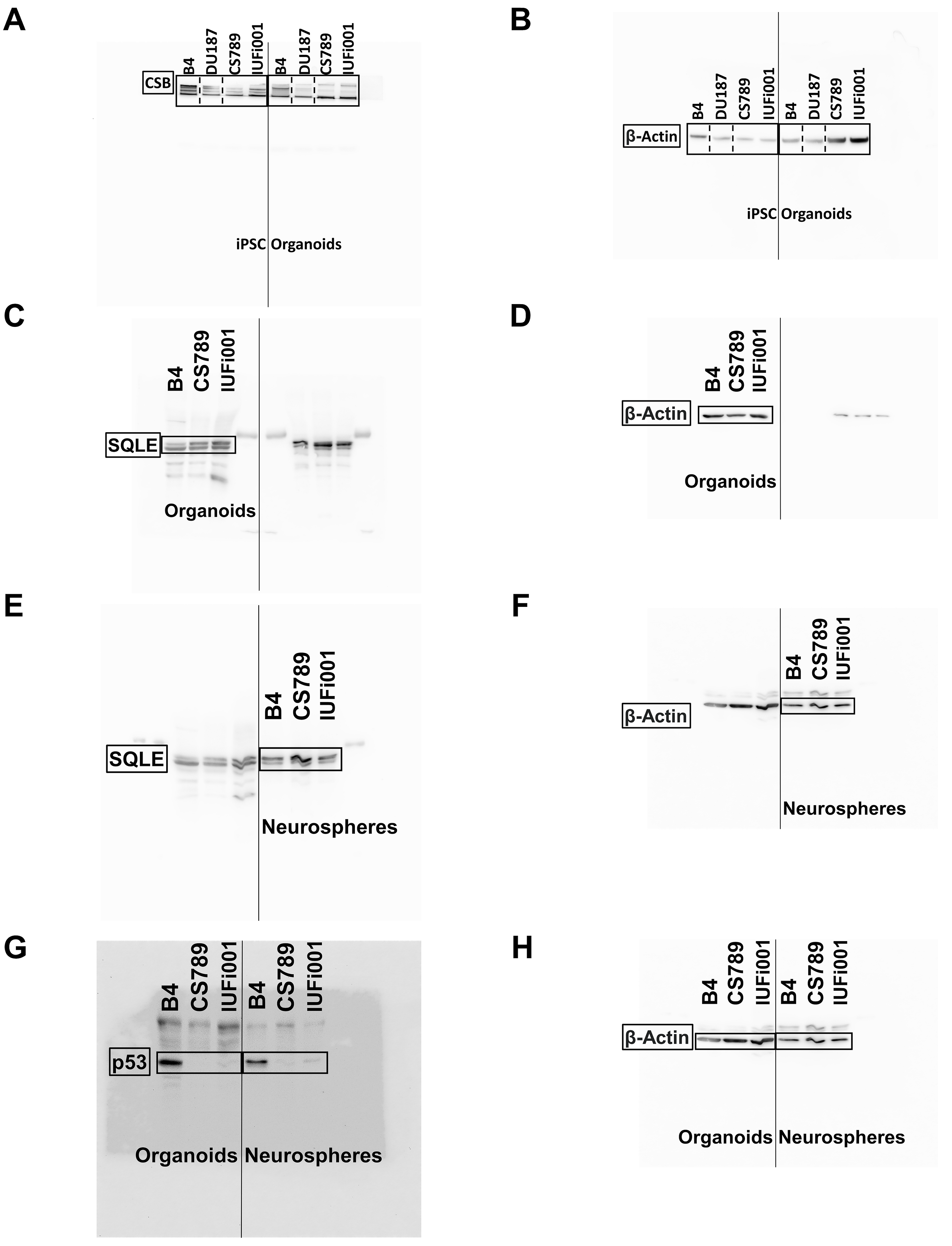

Supplement: Supplementary file 1 [file cells-13-00591-s001.zip › organoids-2917974-supplementary - sub/S3.tif]

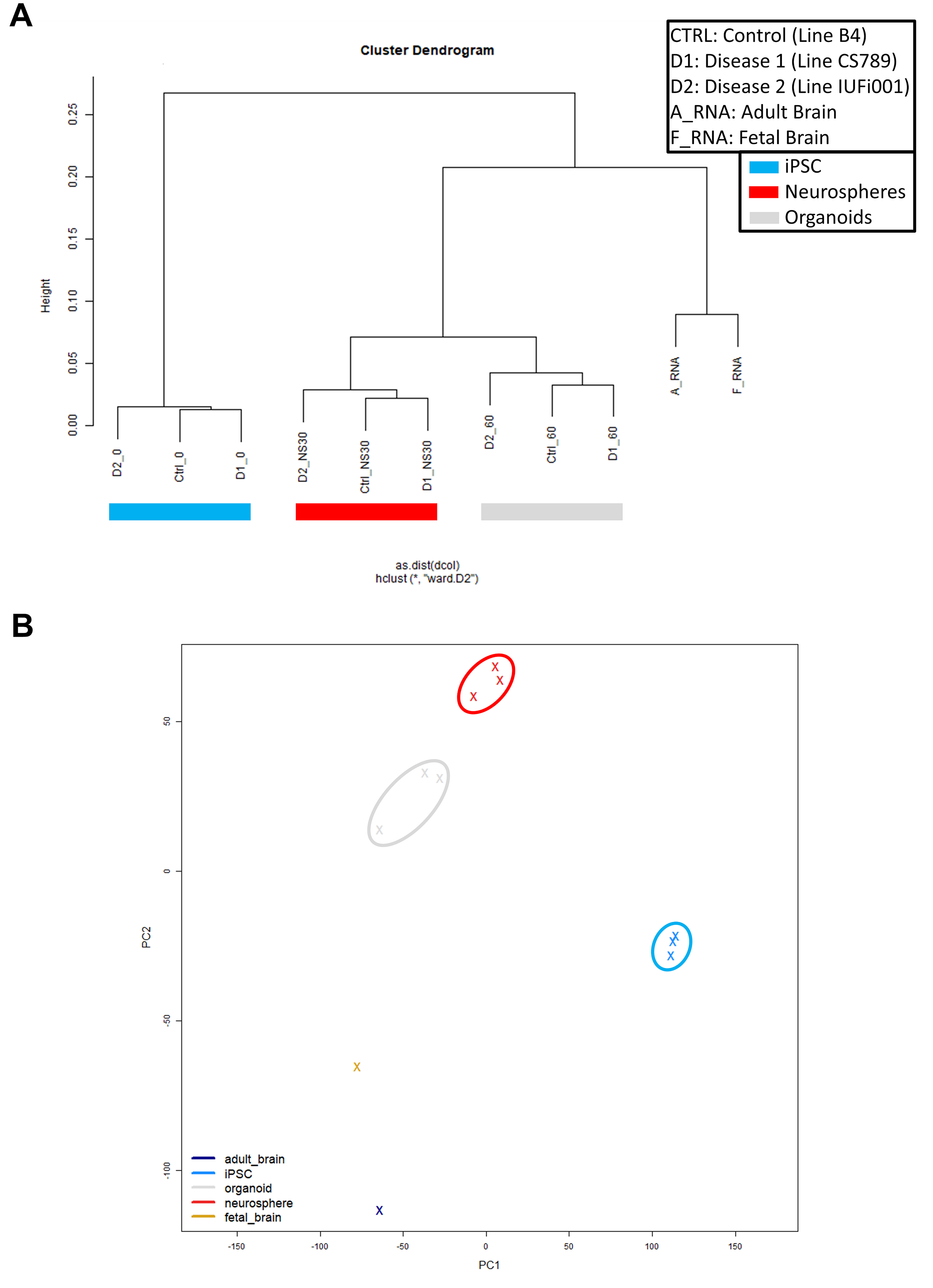

Supplement: Supplementary file 1 [file cells-13-00591-s001.zip › organoids-2917974-supplementary - sub/S4.TIF]

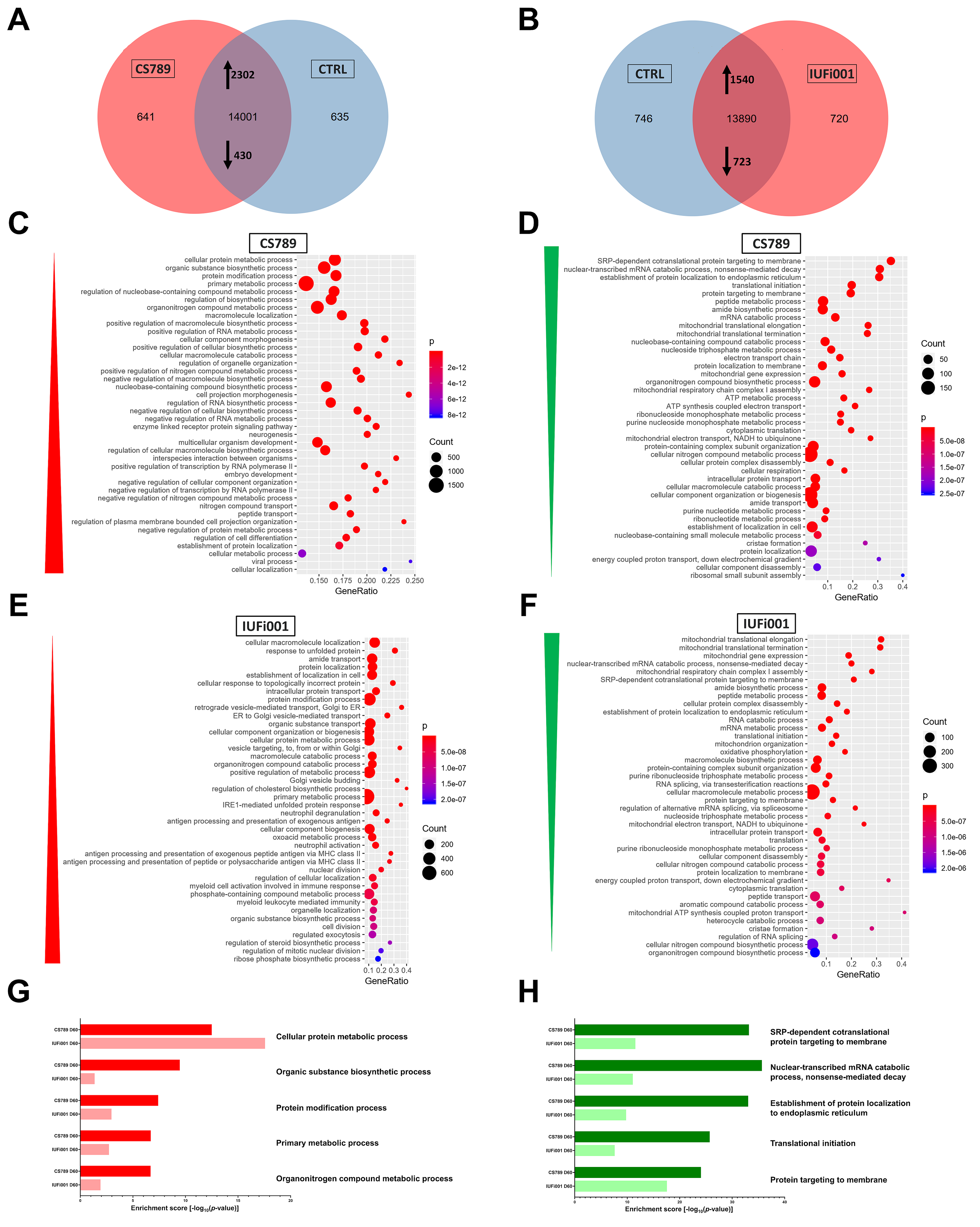

Supplement: Supplementary file 1 [file cells-13-00591-s001.zip › organoids-2917974-supplementary - sub/S5.TIF]

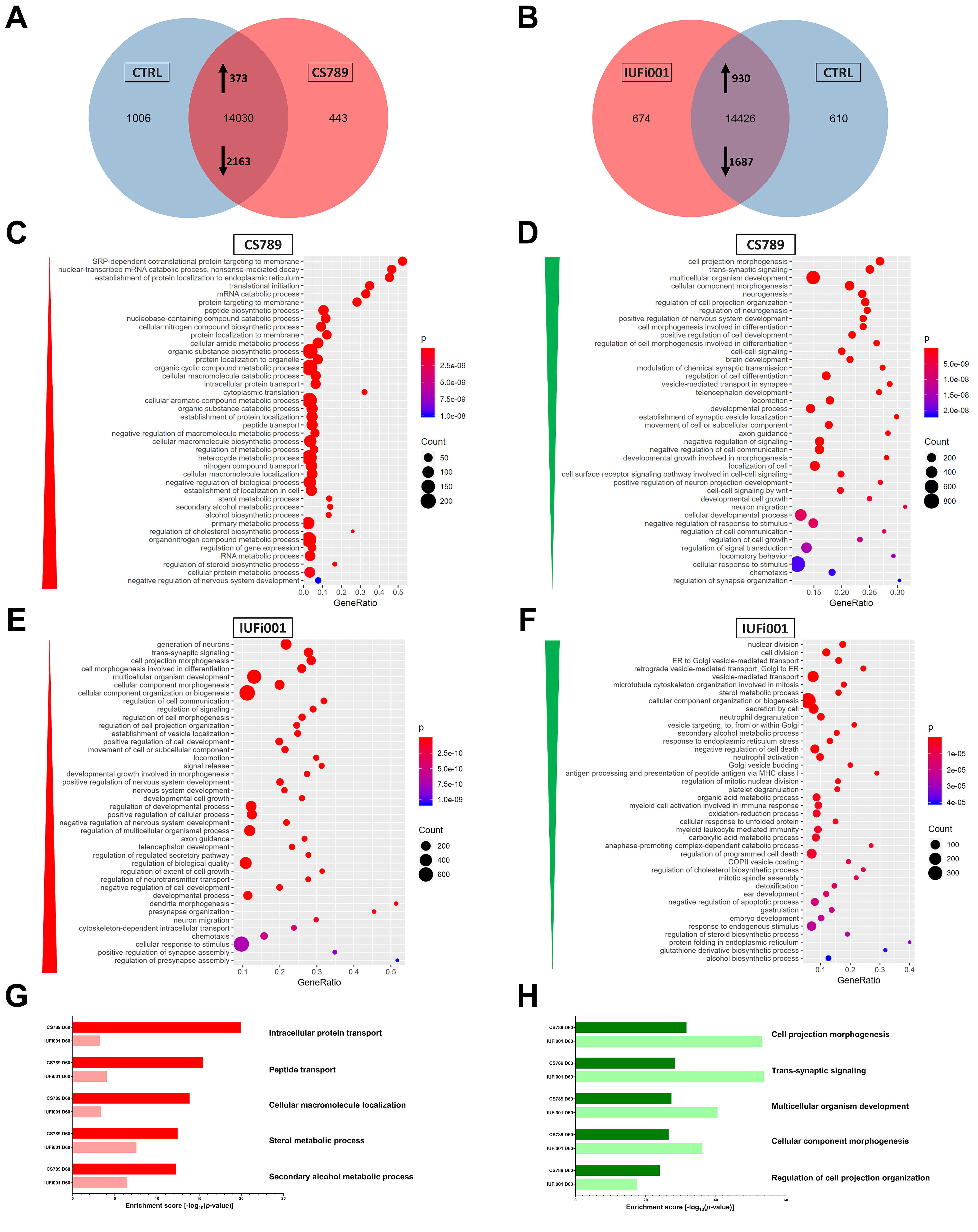

Supplement: Supplementary file 1 [file cells-13-00591-s001.zip › organoids-2917974-supplementary - sub/S6.TIF]

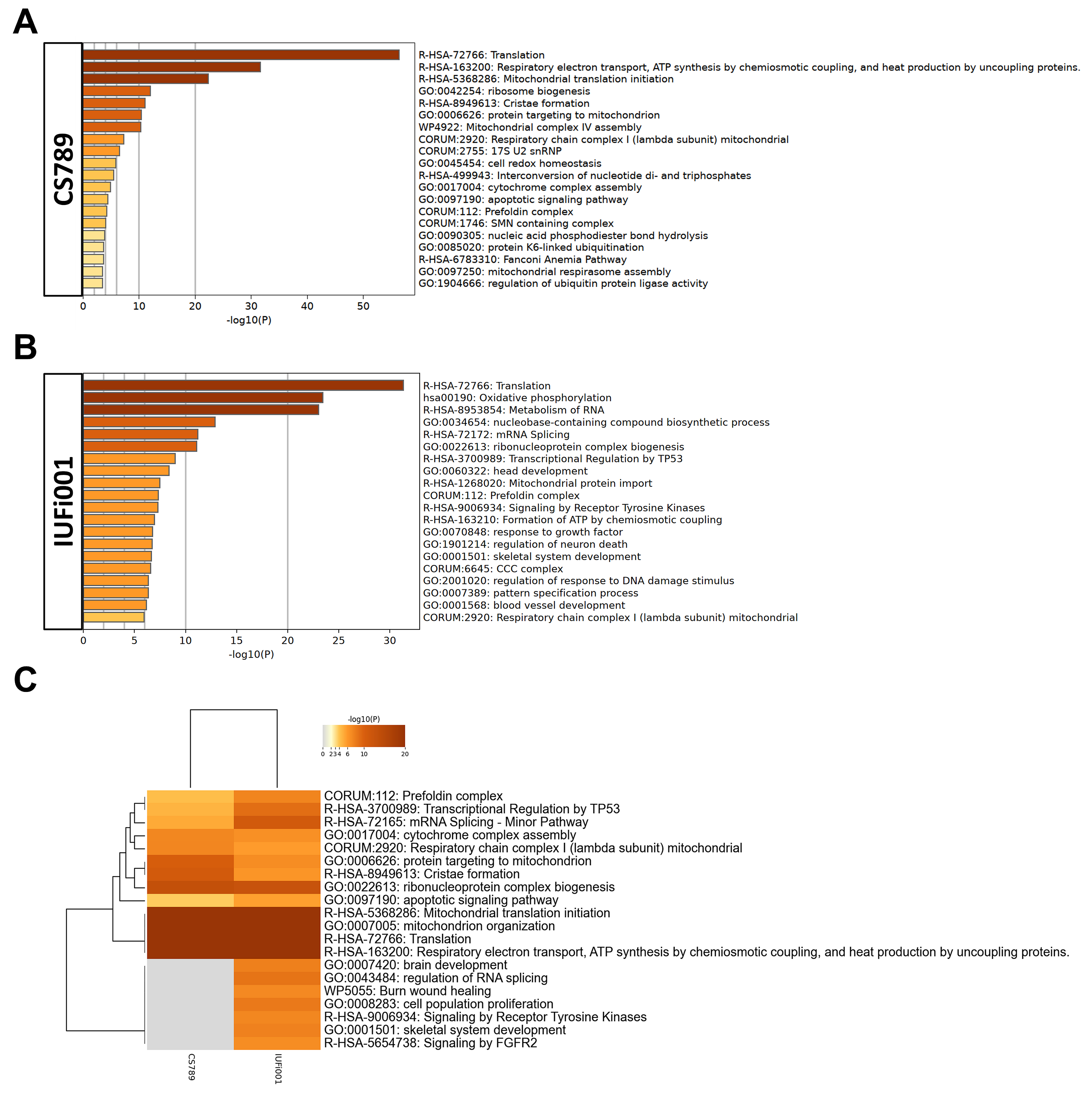

Supplement: Supplementary file 1 [file cells-13-00591-s001.zip › organoids-2917974-supplementary - sub/S7.TIF]

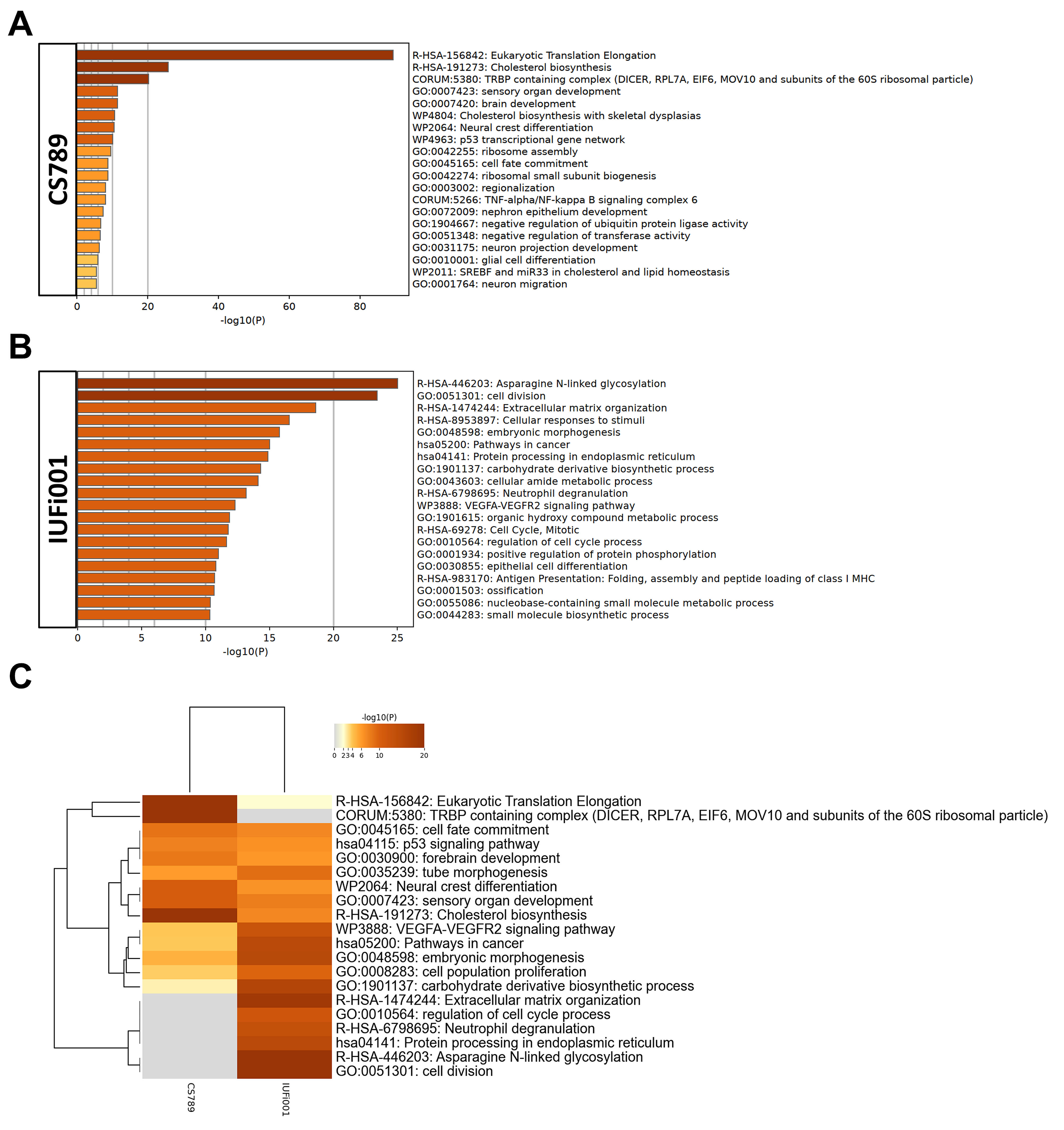

Supplement: Supplementary file 1 [file cells-13-00591-s001.zip › organoids-2917974-supplementary - sub/S8.TIF]

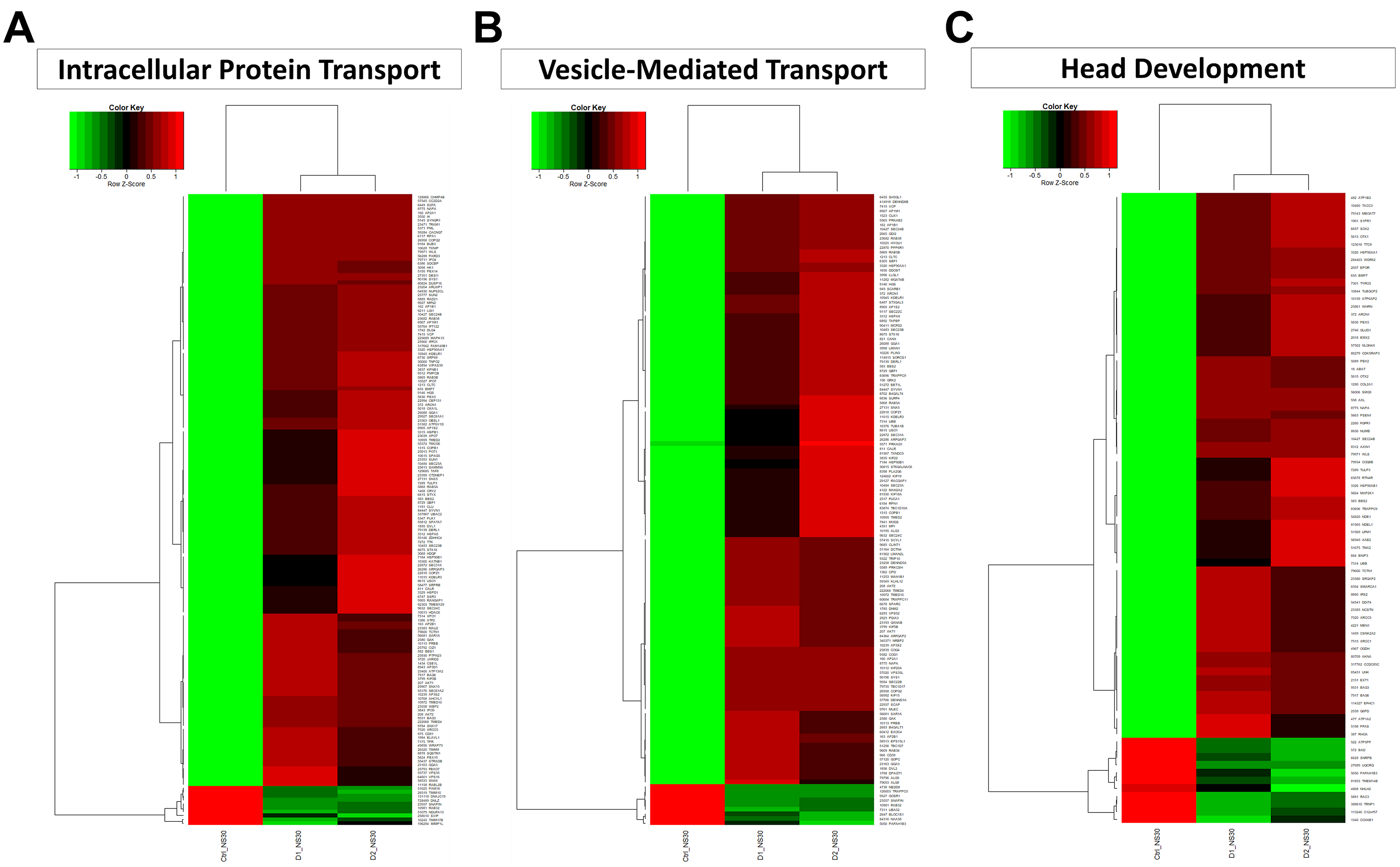

Supplement: Supplementary file 1 [file cells-13-00591-s001.zip › organoids-2917974-supplementary - sub/S9.TIF]
